# Lung and general health effects of Toll-like receptor-4 (TLR4)-interacting SPA4 peptide

**DOI:** 10.1186/s12890-020-01187-7

**Published:** 2020-06-23

**Authors:** Shanjana Awasthi, Negar Rahman, Bin Rui, Gaurav Kumar, Vibhudutta Awasthi, Melanie Breshears, Stanley Kosanke

**Affiliations:** 1 Department of Pharmaceutical Sciences, College of Pharmacy, University of Oklahoma Health Sciences Center, 1110 N. Stonewall Avenue, Oklahoma City, OK 73117 USA; 2 Department of Pharmaceutical Sciences, and Research Imaging Facility, University of Oklahoma Health Sciences Center, Oklahoma City, OK 73117 USA; 3 Department of Veterinary Pathobiology, Oklahoma State University, Stillwater, OK 74078 USA; 4 Division of Comparative Medicine, University of Oklahoma Health Sciences Center, Oklahoma City, OK 73104 USA

**Keywords:** Surfactant protein-A-derived peptide, Pulmonary toxicology, Health effects, Anti-inflammatory activity

## Abstract

**Background:**

A surfactant protein-A-derived peptide, which we call SPA4 peptide (amino acids: GDFRYSDGTPVNYTNWYRGE), alleviates lung infection and inflammation. This study investigated the effects of intratracheally administered SPA4 peptide on systemic, lung, and health parameters in an outbred mouse strain, and in an intratracheal lipopolysaccharide (LPS) challenge model.

**Methods:**

The outbred CD-1 mice were intratracheally administered with incremental doses of SPA4 peptide (0.625–10 μg/g body weight) once every 24 h, for 3 days. Mice left untreated and those treated with vehicle were included as controls. Mice were euthanized after 24 h of last administration of SPA4 peptide. In order to assess the biological activity of SPA4 peptide, C57BL6 mice were intratracheally challenged with 5 μg LPS/g body weight and treated with 50 μg SPA4 peptide via intratracheal route 1 h post LPS-challenge. Mice were euthanized after 4 h of LPS challenge. Signs of sickness and body weights were regularly monitored. At the time of necropsy, blood and major organs were harvested. Blood gas and electrolytes, serum biochemical profiles and SPA4 peptide-specific immunoglobulin G (IgG) antibody levels, and common lung injury markers (levels of total protein, albumin, and lactate, lactate dehydrogenase activity, and lung wet/dry weight ratios) were determined. Lung, liver, spleen, kidney, heart, and intestine were examined histologically. Differences in measured parameters were analyzed among study groups by analysis of variance test.

**Results:**

The results demonstrated no signs of sickness or changes in body weight over 3 days of treatment with various doses of SPA4 peptide. It did not induce any major toxicity or IgG antibody response to SPA4 peptide. The SPA4 peptide treatment also did not affect blood gas, electrolytes, or serum biochemistry. There was no evidence of injury to the tissues and organs. However, the SPA4 peptide suppressed the LPS-induced lung inflammation.

**Conclusions:**

These findings provide an initial toxicity profile of SPA4 peptide. Intratracheal administration of escalating doses of SPA4 peptide does not induce any significant toxicity at tissue and organ levels. However, treatment with a dose of 50 μg SPA4 peptide, comparable to 2.5 μg/g body weight, alleviates LPS-induced lung inflammation.

## Background

Targeting of a receptor or molecule related to disease pathology with a synthetic small molecule or peptide, is an attractive therapeutic approach. However, it is critical that the synthetic molecular entity itself does not induce any significant damage or injury at cellular, tissue, and organ levels. Toll-like receptor 4 (TLR4) is a transmembrane receptor expressed on immune and non-immune cells in all major organs and tissues. The expression of TLR4 is increased at systemic and tissue levels in many disease conditions, including lung infection and inflammation [1]. A TLR4-interacting SPA4 peptide (amino acids: GDFRYSDGTPVNYTNWYRGE) was recently identified [2–4]. The SPA4 peptide binds to activated TLR4 complex and suppresses inflammatory response, but maintains the TLR4-induced uptake, intracellular processing, and clearance of ligands and pathogens [5]. It does not bind to the ligand of TLR4 (Gram-negative bacterial lipopolysaccharide, LPS) or Gram-negative bacteria directly [5, 6]. Intraperitoneally and intratracheally administered SPA4 peptide was recently demonstrated to reduce inflammation, bacterial burden, and associated lung injury in mouse models of LPS-induced lung inflammation and *Pseudomonas aeruginosa*-induced lung infection, respectively [4, 5].

The present study investigated the effects of escalating doses of intratracheally administered SPA4 peptide on tissues and organs, and general health parameters in CD-1 mouse model, and biological activity of SPA4 peptide in intratracheally LPS challenged C57BL6 mice.

The outbred CD-1 mice were included which exhibit genetic diversity similar to that found within and between human populations [7]. The CD-1 mice included in this investigation have been used extensively for studies related to safety and toxicity assessment of new drugs and chemical entities [8–11]. Previously published results have demonstrated that the SPA4 peptide treatment on its own does not induce any cytokine response in dendritic cells and macrophages [2, 4, 12]. However, the toxicity of SPA4 peptide remained unknown. The results of this study demonstrate that repeated intratracheal administration of the SPA4 peptide does not induce any symptom of sickness or mortality, and does not affect body weights or tissue architecture of major organs, including lung. The lung wet/dry weight ratio (as a measure of pulmonary edema), biochemical markers of lung injury and inflammation (levels of lung protein, albumin, and lactate, and activity of lactate dehydrogenase [LDH]), serum biochemistry parameters, and blood gas and electrolytes also remain unaffected. There was no evidence of SPA4 peptide-specific IgG antibody response in SPA4 peptide-treated mice. These results provide an initial toxicity assessment of repeated intratracheal administration of SPA4 peptide in a mouse model. Furthermore, the findings demonstrate that therapeutically administered SPA4 peptide via intratracheal route suppresses lung inflammation in an intratracheal LPS challenge model in mice. Together, these results point towards therapeutic efficacy of SPA4 peptide without any significant toxicity at tissue and organ levels.

## Methods

### SPA4 peptide

SPA4 peptide (amino acids: GDFRYSDGTPVNYTNWYRGE) was synthesized at Genscript, NJ. The purity of each batch of the peptide was confirmed by mass spectroscopy and high-performance liquid chromatography (HPLC). The stock solutions of the SPA4 peptide were prepared in endotoxin-free water.

### Mice

Five-to-six-week-old female CD-1 and C57BL6 mice were purchased from Charles River Laboratories (Wilmington, MA) and Jackson Laboratory (Bar Harbor, ME), respectively. Mice were acclimated for at least 1 week before their inclusion in an experiment. Mice were randomly assigned to a study group in separate experiments performed on different occasions. Up to five mice assigned to a particular group were housed together. Mice were given food and water ad libitum throughout the study. The animal studies were approved by the Institutional Animal Care and Use Committee (Protocol numbers: 12–105-HI, 15–075-HI, 17–081-CHR) at the University of Oklahoma Health Sciences Center (OUHSC), Oklahoma City, OK, USA.

### Intratracheal administration of escalating doses of SPA4 peptide in CD-1 mice

Mice were weighed, anesthetized using isoflurane followed by ketamine/xylazine, and intratracheally administered with escalating doses of SPA4 peptide suspended in endotoxin-free water (0.625, 1.25, 2.5, 5, and 10 μg/g body weight) every 24 h for 3 days. Mice were weighed daily and monitored for symptoms of sickness (ruffled fur, eye exudate, diarrhea, prostration, reactivity to tail-hold stimuli), per the described method [13]. The instilled volume of SPA4 peptide was adjusted daily as per the body weight. Separate groups of mice were given an equivalent volume of endotoxin-free water (vehicle) or were left untreated. At the end of the study, all mice were anesthetized with isoflurane, euthanized by cervical dislocation, and necropsied after 24 h of last administration. At the time of necropsy, blood, major organs (liver, heart, kidney, spleen, and intestine), and whole lung or lung tissue samples were collected.

### Collection and processing  of blood, major organs, including lung, and lung tissues

Blood samples were collected by cardiac puncture and immediately subjected to the measurement of blood gas and electrolytes. The remaining blood samples were clotted for serum separation.

Major organs (liver, heart, kidney, spleen, and intestine) were harvested from the necropsied mice, fixed in 10% formalin, and processed for histology. Lung tissue pieces were snap-frozen in liquid nitrogen, or were fixed in 10% formalin for histological processing. The harvested whole lungs from a separate set of mice were weighed and dried to obtain lung wet/dry weight ratios.

### Assessment of biochemical markers of lung injury (levels of total protein, albumin, and lactate; lung wet/dry weight ratios; and LDH activity)

Lung tissue pieces were homogenized in homogenization buffer containing 1% Igepal CA630, 0.1% sodium dodecyl sulfate, 0.5% sodium deoxycholate, 0.5 mM disodium ethylenediamine tetraacetic acid (EDTA, pH 8.0), 0.5 mg/ml leupeptin, 0.7 mg/ml pepstatin, 200 μM phenylmethylsulfonyl fluoride, 200 μM sodium orthovanadate, and 10 μM sodium fluoride, and were processed as described earlier [14, 15]. The levels of total protein, albumin, and lactate, and activity of LDH were determined in lung tissue homogenates. All of these parameters have been used previously as measures of lung inflammation, hypoxia, and injury [16, 17].

Total protein concentration was measured in diluted lung tissue homogenates against bovine serum albumin (BSA) standard solutions using a bicinchoninic acid (BCA) assay kit (Thermo Fisher Scientific, MA). The levels of albumin were measured in 1:5000 diluted lung tissue homogenates using a commercially available enzyme-linked immunosorbent assay (ELISA) kit (Bethyl Labs, TX), as described earlier [16]. The levels of lactate were measured in diluted lung tissue homogenate samples using a fluorometric assay kit (Biovision, CA), per the manufacturer’s instructions, as described earlier [16]. The concentrations of total protein, albumin, and lactate were normalized with g wet lung weight.

In separate experiments, whole lungs were harvested. The wet lung weights were measured. Lungs were then dried at 60°C for 48 h in a vacuum oven (Fisher Scientific, PA) and weighed to obtain dry lung weight. Lung wet-to-dry weight ratios (sign of lung edema) were calculated and compared among different groups of mice.

As an additional marker of cytotoxicity and tissue injury, the LDH activity was measured in lung tissue homogenates of CD-1 mice in a kinetic manner, per the manufacturer’s instructions (Abcam, MA). In this assay, the LDH reduces nicotinamide adenine dinucleotide (NAD) to NADH, which is detected by incubating with a specific probe. Briefly, NADH standard solutions (5–40 nmol/well) and diluted lung tissue homogenates (50 μl total volume per well) were mixed with 50 μl of reaction mix of assay buffer and substrate solution. The absorbance readings were taken at 450 nm on a Biotek microplate reader in a kinetic mode, every 3 min for 4 h at 37°C protected from light. The nmol amounts of NADH (B) generated by LDH were obtained in lung tissue homogenate samples by measuring the difference in the absorbances at two time points (∆T) in the linear range of the standard curve. The LDH activity (nmol/min/ml) was then calculated as: [(B/∆T x ml sample volume added into the reaction well) x sample dilution factor]. One unit of LDH activity is the amount of enzyme that catalyzes the conversion of lactate to pyruvate to generate 1 μmol or NADH per min at pH 8.8 at 37°C. Assay-specific positive and negative controls were included each time, per the manufacturer’s instructions. The levels of LDH activity were normalized with mg total lung protein.

### Tissue histology

Formalin-fixed randomly collected lung tissues and other organs from CD-1 mice were transferred into 70% ethanol after 24 h. Fixed tissue specimens or whole lungs were dehydrated in ethanol, infiltrated, embedded into paraffin, sectioned, and stained with hematoxylin and eosin (H&E) stain (Precision Histology, Oklahoma City, OK; Oklahoma State University, Stillwater, OK; and Stephenson Cancer Center Histology Core Laboratory, OUHSC, Oklahoma City, OK). The H&E-stained tissues were then independently examined by board-certified veterinary pathologists in a blinded manner.

### Blood gas and electrolyte analysis

About 200 μl of freshly collected blood was injected in the VetStat Electrolyte and Blood Gas Analyzer (IDEXX Laboratories, Inc., ME). Read-outs were gathered for blood gas and electrolytes: partial pressure carbon dioxide (pCO_2_), total carbon dioxide (tCO_2_), partial pressure oxygen (pO_2_), hydrogen ion concentration (pH), anion gap (AG), base excess (BE), total hemoglobin concentration (tHb), sodium (Na^+^), potassium (K^+^), chloride (Cl^−^), and bicarbonate (HCO_3_^−^). The remainder of the blood was clotted at room temperature and centrifuged for the collection of serum. Serum samples were stored frozen until further analysis.

### Serum biochemistry profile of SPA4 peptide-treated CD-1 mice

Frozen serum samples were analyzed at IDEXX Bio Analytics Pathology Services, CA, for the following biochemical markers of toxicity and metabolic and physiological abnormalities: alkaline phosphatase (ALP), aspartate aminotransferase (AST), alanine aminotransferase (ALT), creatine kinase, gamma-glutamyl transferase (GGT), albumin, total bilirubin, globulin, total protein, blood urea nitrogen (BUN), creatinine, cholesterol, glucose, and bile acids.

### SPA4 peptide-specific total IgG response in CD-1 mice treated with SPA4 peptide

Antibody was raised in rabbit against SPA4 peptide, and was affinity purified (Thermo Fisher Scientific, MA). An ELISA method was developed using SPA4 peptide and affinity-purified antibody. Briefly, microwell strips were coated with 0.1 μg SPA4 peptide or BSA per well diluted in 0.1 M NaHCO_3_, pH 9.6, overnight at 4°C and at room temperature for 1 h. Nonspecific sites were blocked using 1% BSA in Tris buffered saline containing 0.05% Tween 20 (TBST), for 1 h at 30°C. The microwells were then washed with TBST and incubated overnight with 1:10, 1:100, 1:1000 diluted mouse serum samples or 1:2000 diluted affinity purified SPA4 peptide-specific antibody (1.34 mg/ml, positive control) at 4°C. After washing with TBST, the microwells were then incubated with 1:2000 diluted alkaline phosphatase-conjugated anti-mouse IgG (whole molecule) or anti-rabbit IgG (whole molecule) antibody at 30°C for 1 h. Finally, the immune complexes were detected by using p-nitrophenyl phosphate solution (Sigma-Aldrich, MO). The reaction was stopped by the addition of 30 μl of 3 M NaOH, and the optical density (OD) was read at 405 nm spectrophotometrically. The ∆OD405 readings were obtained by subtracting the reading obtained for BSA-coated wells from those obtained from SPA4 peptide-coated wells for the mouse serum samples and positive control.

### SPA4 peptide activity in a mouse model of LPS-induced lung inflammation

We also included an intratracheal LPS challenge model. C57BL6 mice were weighed and anesthetized using isoflurane followed by administration of ketamine and xylazine. Anesthetized mice were then intratracheally instilled with highly-purified, low-protein, *Escherichia coli* O111:B4-derived LPS (Calbiochem, San Diego, CA; 5 μg/g body weight), per the method described earlier [5]. After 1 h of LPS challenge, mice were treated with 50 μg of SPA4 peptide or an equivalent amount of vehicle via intratracheal route, or were left untreated. The instilled volume of SPA4 peptide or vehicle was kept same. At the end, after 4 h of LPS challenge, mice were observed and graded for symptoms of sickness as described above. Mice were then euthanized and necropsied. At the time of necropsy, blood, and whole lung or lung tissues were harvested. Blood gas and electrolytes, and levels of total protein, albumin, and lactate, were determined in blood and lung tissue homogenates, respectively, as described above. Levels of lactate were also measured in serum samples. Whole lungs harvested from separate mice were utilized for determining lung wet/dry weight ratios or histological changes.

### Lung histology in LPS-challenged and SPA4 peptide treated mice

Whole lungs were fully inflated using 10% formalin, immersed, and were kept at room temperature for 24 h. The lungs were then transferred and kept in 75% ethanol until further processing for histopathology. Tissues were dehydrated through 70–100% alcohol baths, cleared with xylene, and finally infiltrated with and embedded into paraffin (Precision Histology, Oklahoma City, OK). Lung tissue sections (5 μm in thickness) were stained with hematoxylin and eosin (H&E) and examined for inflammation by a board-certified veterinary pathologist.

Lung inflammation was semi-quantitatively graded based on four different criteria, as following: (A) extent of peribronchial/bronchiolar infiltrates (% of airways affected; 0 = none, 1 < 25%, 2 = 25–49%, 3 = 50–75%, 4 > 75%); (B) severity of peribronchial/ bronchiolar infiltrates (estimated average of affected airways; 0 = none, 1 = few individual inflammatory cells, 2 = multifocal aggregates of inflammatory cells, 3 = segmental or partial cuff of inflammatory cells, 4 = complete cuff of inflammatory cells); (C) extent of alveolar infiltrates (% of interbronchial parenchyma affected)-count quadrants for 10 (20X fields) (0 = none, 1 = one quadrant, 2 = two quadrants, 3 = three quadrants, 4 = four quadrants); and (D) severity of alveolar septal and luminal neutrophilic infiltrates (score one 40x field from most severely affected region of each of 20 x fields examined for quadrant counts (above) (0 = none, 1 = individual neutrophils,< 10 per high-power field (HPF), 2 = few neutrophilic aggregates, < 3 aggregates/HPF, 10–30 neutrophils/HPF, 3 = moderate neutrophilic aggregates, 4–7 aggregates/HPF, 40–70 neutrophils/HPF, 4 = severe neutrophilic aggregates, > 7 aggregates/HPF, > 70 neutrophils/HPF). An overall score for pulmonary inflammation was obtained by adding the individual numerical grades for parameters (A-D).

In order to assess the cellularity, we obtained five random photomicrographs (one representative of a lobe) of H&E-stained lung tissue sections. The images were uploaded onto the STEPanizer program to draw the regions of interest and to count the number of cells [18]. The dimensions for the randomly selected regions of interest were kept the same for each photomicrograph and the nucleated cells were counted.

The number of intercepts were counted as a measure of changes in the air space sizes. The photomicrographs were taken using a 25x objective lens, and were uploaded onto the STEPanizer program. Equidistant lines were plotted in horizontal and vertical directions. Images with bronchi, large airways, and blood vessels were excluded from the measurements. The number of intercepts were counted on horizontal and vertical lines. Since the 10% formalin fixation and paraffin embedding of mouse lungs have only a small effect on linear dimension, a correction factor was not included [19].

### Statistical analysis

Data were analyzed using one-way or two-way Analysis of Variance (ANOVA) followed by Tukey’s post hoc analysis for multiple comparisons (GraphPad Prism software, San Diego, CA). Statistical significance was defined as a *p* value of < 0.05.

## Results

### Symptoms of sickness and body weights of SPA4 peptide-treated CD-1 mice

**Fig. 1:**
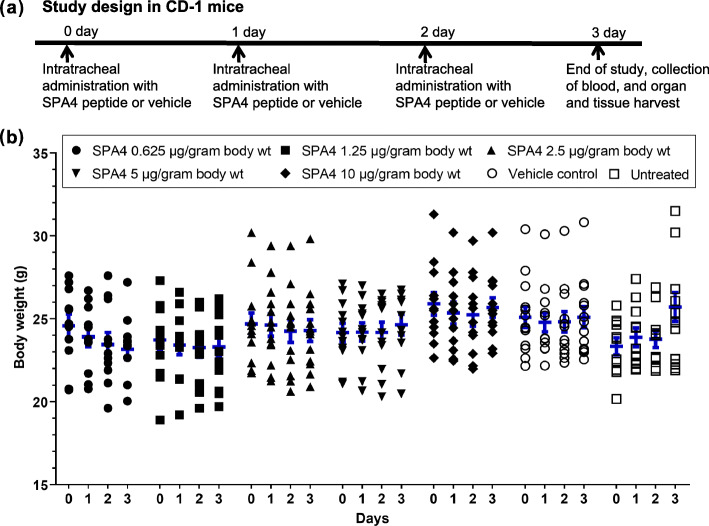
Assessment of body weight in SPA4 peptide-treated CD-1 mice. The schedule of SPA4 peptide treatment and study design are illustrated in (**a**). Body weights were taken for each mouse after every 24 h. Results shown are from 11 to 15 mice per group (seven groups total) (**b**). No statistically significant difference was observed in body weights of mice over time and among different study groups (Two-way ANOVA followed by Tukey’s post hoc analysis)

All SPA4 peptide-treated, untreated, and vehicle-treated CD-1 mice survived and showed no sign of sickness. Mice remained fully active during the study period. Correspondingly, no statistically significant change was noted in the body weights of SPA4 peptide-treated, untreated, or vehicle-treated mice over time (maximum 8% change in body weights; Fig. 1).

### Intratracheal administration of SPA4 peptide and markers of lung injury or edema in CD-1 mice

**Fig. 2:**
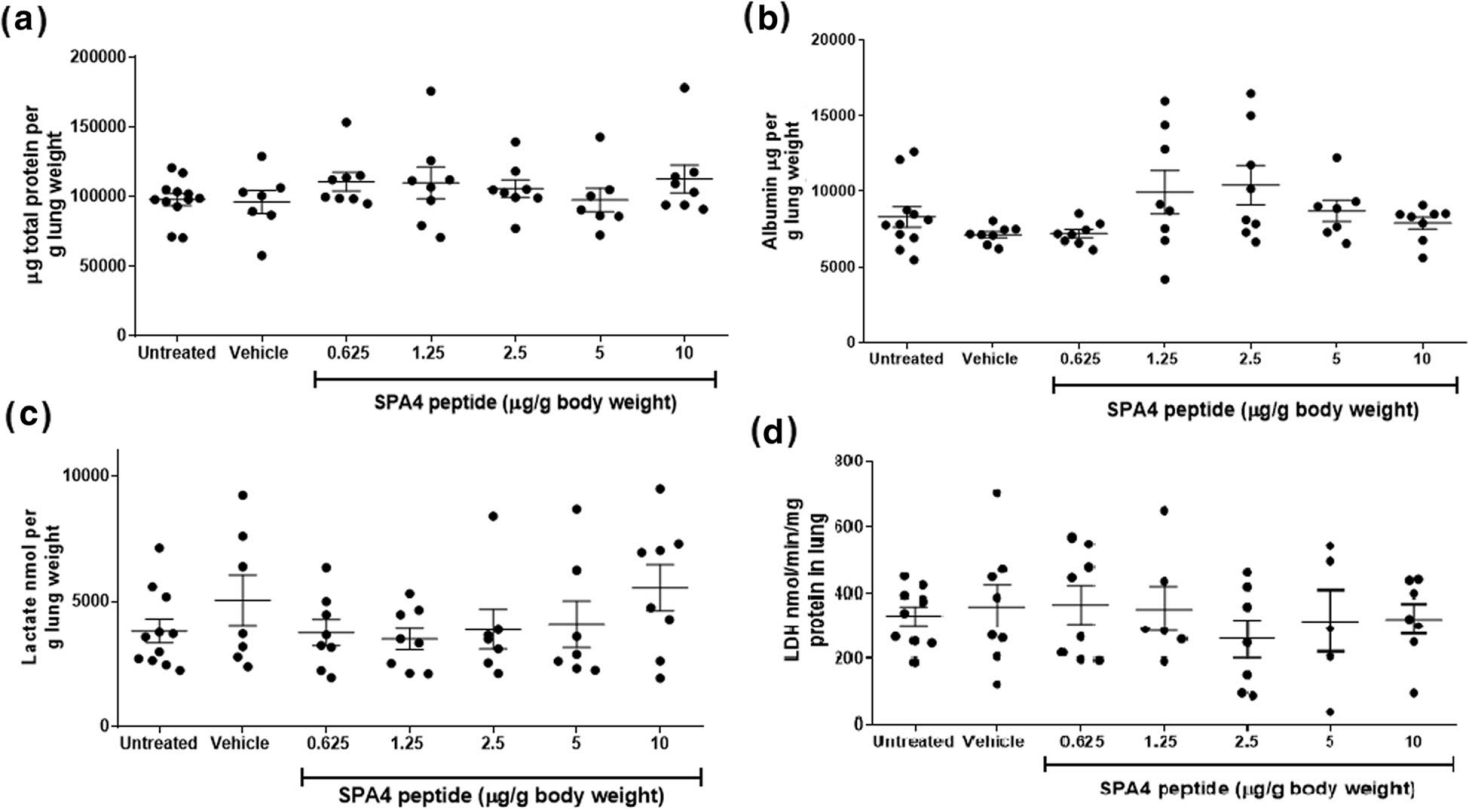
Levels of total lung protein, albumin, and lactate, and LDH activity. Lung tissue homogenates were subjected to the measurement of total protein by BCA assay (**a**), albumin by ELISA (**b**), lactate by a fluorometric assay (**c**), and LDH activity using a colorimetric method (**d**). The amounts of total protein, albumin, and lactate, and activity of LDH, were normalized with gram lung weight or total lung protein. Results shown are mean + *SEM* of measurements in lung tissue homogenates from 5 to 12 CD-1 mice per group (seven groups total). No statistically significant difference was observed in the measurement between the study groups (One-way ANOVA followed by Tukey’s post hoc analysis)

Results indicate that treatment of CD-1 mice with escalating doses of SPA4 peptide did not affect the amounts of total lung protein, albumin, and lactate, LDH activity, or lung edema. Total protein concentration was measured against BSA using the BCA assay kit (sensitivity 7.81 μg/ml). The amounts of total protein (range of mean values 96,024–112,564 μg/g lung) were not significantly different among groups (Fig. 2a). Total albumin was measured using ELISA (sensitivity 1.23 ng/ml). The albumin concentration (range of mean values 7123–10,400 μg/g lung) did not differ among groups (Fig. 2b). Levels of lactate (range of mean values 3815–5537 nmol/g lung) were unaffected (Fig. 2c). The LDH activity, denoted as nmol of NADH generated/min/ml, was determined in a kinetic assay, and was normalized with mg total lung protein. The LDH activity (range of mean values 261–366 nmol/min/ml per mg total lung protein) was not significantly different in the lung tissues harvested from mice in different groups (Fig. 2d).

**Fig. 3:**
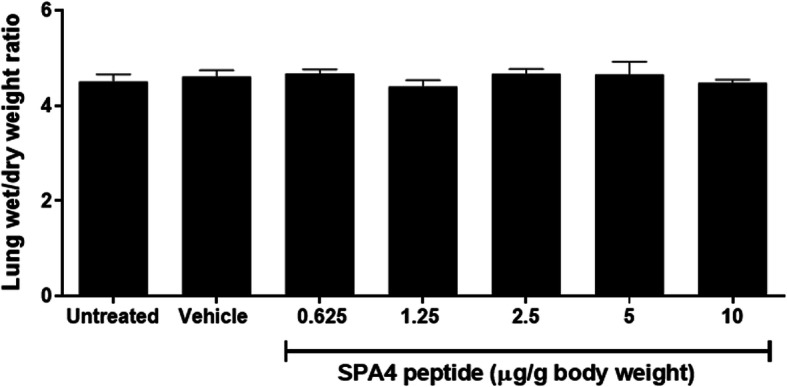
Assessment of lung wet/dry weight ratio as an indicator of lung edema. Whole lungs harvested from CD-1 mice were weighed immediately (wet weight). After drying at 60°C for 48 h, lungs were weighed again. The ratios of lung wet/dry weights were calculated. Data shown are mean + *SEM* values from lungs of 3–5 mice per group (seven groups total). There was no statistically significant difference in the values among different study groups (One-way ANOVA followed by Tukey’s post hoc analysis)

There was no sign of lung edema estimated by lung wet/dry weight ratios (range of mean values 4.1–5.1) among different groups (Fig. 3). These results provide first-time evidence that the SPA4 peptide is not toxic or injurious to the lung. All biochemical assays and ELISA were performed in a blinded manner.

### Histological findings from harvested lung, heart, kidney, spleen, liver, and intestine

**Fig. 4:**
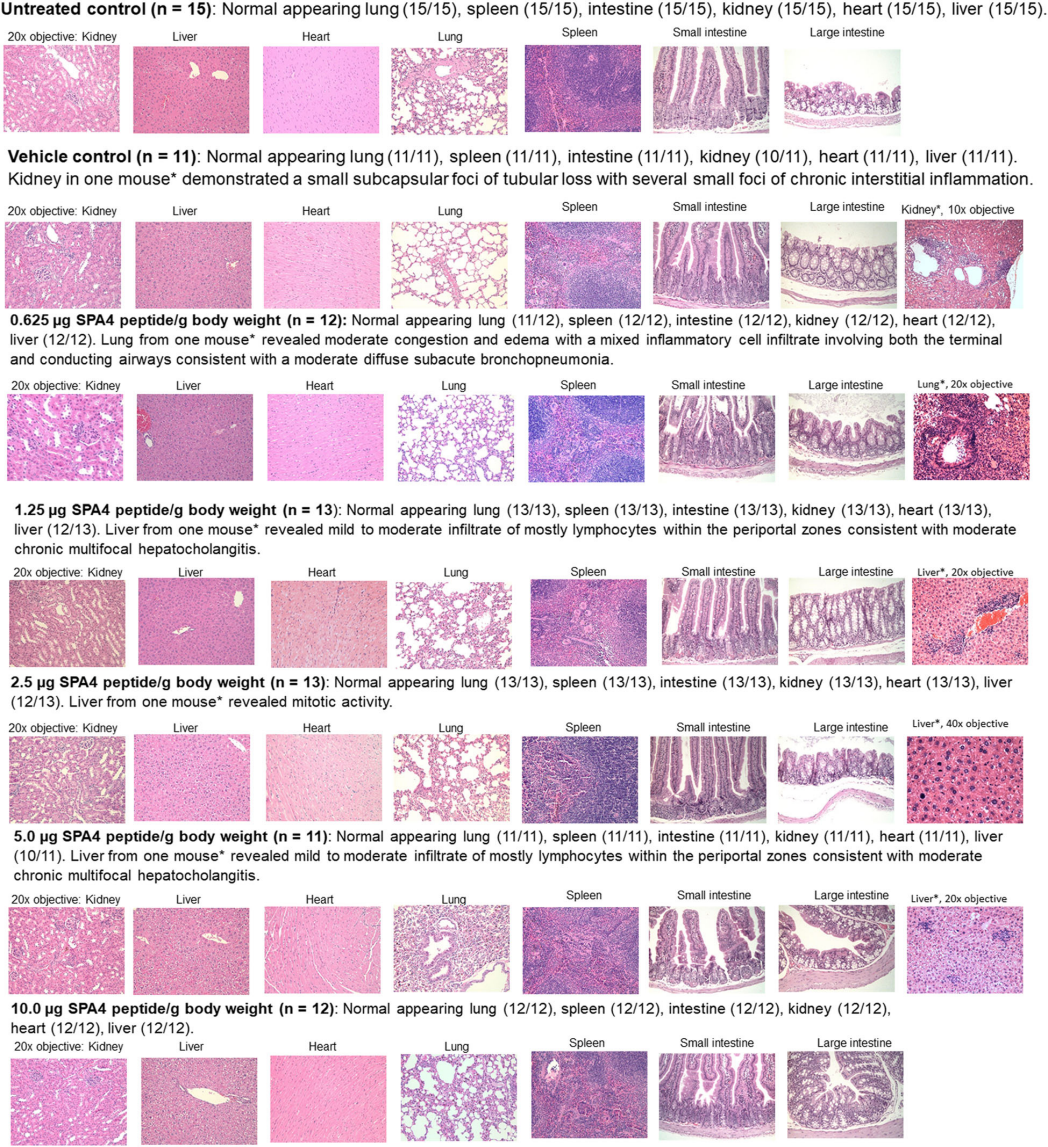
Histological evaluation of H&E-stained sections of tissues (lung, heart, kidney, liver, spleen, and intestine) harvested from SPA4 peptide-treated, vehicle-treated, and untreated CD-1 mice. Major organs were harvested from mice at the time of necropsy and fixed in 10% buffered-formalin overnight, dehydrated, embedded in paraffin, sectioned, and stained with H&E. Tissue sections were evaluated for any evidence of tissue damage and inflammation by board-certified veterinary pathologists. Representative photomicrographs are shown from respective tissue section for each study group (11–15 mice per group, seven groups total) revealing normal histology. Atypical findings in isolated specimens (shown as*) from one mouse in selected study groups are shown, which are not related to the dose of SPA4 peptide or study design

The board-certified veterinary pathologists independently examined the H&E-stained tissue sections of kidney, liver, heart, lung, spleen, and intestine harvested from mice that were treated with five escalating doses of SPA4 peptide, treated with vehicle, or left untreated (*n* = 11–15 per group). In the majority of mice, kidneys appeared normal, with no evidence of vascular changes, glomerular or tubular damage, or acute inflammation. A kidney in one vehicle-control mouse revealed a small subcapsular foci of tubular loss with several small foci of chronic interstitial inflammation. Lung in majority of the mice in different groups revealed no vascular change, terminal or conducting airway damage, or acute inflammation. However, lung tissue from one mouse treated with 0.625 μg SPA4 peptide/g body weight revealed moderate congestion and edema with a mixed inflammatory cell infiltrate involving both the terminal and conducting airways. This is consistent with a moderate diffuse subacute bronchopneumonia (Fig. 4, Table 1).

**Table 1:** Histological findings in H&E-stained tissue specimens of CD-1 mice intratracheally treated with different doses of SPA4 peptide (*n* = 11–15 mice per group)

Group	Histological findings
0.625 μg SPA4 peptide/g body weight (*n* = 12)	Normal-appearing lung (*n* = 11/12), spleen (*n* = 12/12), intestine (*n* = 12/12), kidney (*n* = 12/12), heart (*n* = 12/12), and liver (*n* = 12/12). Lung tissue from one mouse revealed moderate congestion and edema with a mixed inflammatory cell infiltrate involving both the terminal and conducting airways, consistent with a moderate diffuse subacute bronchopneumonia.
1.25 μg SPA4 peptide/g body weight (*n* = 13)	Normal-appearing lung (*n* = 13/13), spleen (*n* = 13/13), intestine (*n* = 13/13), kidney (*n* = 13/13), heart (*n* = 13/13), and liver (*n* = 12/13). Liver from one mouse revealed a mild to moderate infiltrate of mostly lymphocytes within the periportal zones consistent with moderate chronic multifocal hepato-cholangitis.
2.5 μg SPA4 peptide/g body weight (*n* = 13)	Normal-appearing lung (*n* = 13/13), spleen (*n* = 13/13), intestine (*n* = 13/13), kidney (*n* = 13/13), heart (*n* = 13/13), and liver (*n* = 12/13). Liver from one mouse revealed increased mitotic activity.
5 μg SPA4 peptide/g body weight (*n* = 11)	Normal-appearing lung (*n* = 11/11), spleen (*n* = 11/11), intestine (*n* = 11/11), kidney (*n* = 11/11), heart (*n* = 11/11), and liver (*n* = 10/11). Liver tissue from one mouse revealed mild to moderate infiltration of mostly lymphocytes within the periportal zones consistent with moderate chronic multifocal hepato-cholangitis.
10 μg SPA4 peptide/g body weight (*n* = 12)	Normal-appearing lung (*n* = 12/12), spleen (*n* = 12/12), intestine (*n* = 12/12), kidney (*n* = 12/12), heart (*n* = 12/12), and liver (*n* = 12/12).
Vehicle control (*n* = 11)	Normal-appearing lung (*n* = 11/11), spleen (*n* = 11/11), intestine (*n* = 11/11), kidney (*n* = 10/11), heart (*n* = 11/11), and liver (*n* = 11/11). The kidney in one mouse revealed a small subcapsular foci of tubular loss with several small foci of chronic interstitial inflammation.
Untreated control (*n* = 15)	Normal-appearing lung (*n* = 15/15), spleen (*n* = 15/15), intestine (*n* = 15/15), kidney (*n* = 15/15), heart (*n* = 15/15), and liver (*n* = 15/15).

Liver tissues appeared normal in the majority of mice, with no evidence of vascular changes, hepatocellular damage, or acute inflammation with only an occasional mitotic figure. Liver tissue from one mouse treated with 1.25 or 5 μg SPA4 peptide/g body weight revealed a mild to moderate infiltrate of mostly lymphocytes within the periportal zones, consistent with moderate chronic multifocal hepato-cholangitis. Liver tissue from one mouse treated with 2.5 μg SPA4 peptide/g body weight revealed increased mitotic activity (Fig. 4, Table 1). These hepatic changes in isolated specimens were not related to the dose of SPA4 peptide or any of the biochemical or physiological parameter.

The heart appeared normal revealing no evidence of vascular change, myofiber damage, or acute inflammation.

The intestines revealed no evidence of mucosal damage, or increased crypt epithelial mitotic activity.

The spleen from all mice revealed no evidence of vascular change, lymphoid follicular damage, or acute inflammation (Fig. 4, Table 1).

### Effect of SPA4 peptide treatment on blood gas and electrolytes

Blood gas and electrolyte parameters were not significantly different in SPA4 peptide-treated mice compared with untreated mice or vehicle-treated controls. Mean blood pH varied from 7.19 to 7.42 among different groups, and was not significantly different. Measured levels of partial pressure of oxygen (pO_2_), carbon dioxide (pCO_2_), and total carbon dioxide (tCO_2_), were not significantly different among groups, indicating no sign of hyperoxia, hypoxia, acidosis, or alkalosis.

A balance of electrolytes is critical for the normal functioning of cells and organs. Measured levels of indicators of normal cellular function, tHb, Na^+^, K^+^, HCO_3_^−^, and Cl^−^, were not significantly different in SPA4 peptide-treated mice compared with those in healthy untreated and vehicle-treated mice (Table 2).

**Table 2:** Blood gas and electrolyte measurements in fresh blood harvested from CD-1 mice intratracheally administered with different doses of SPA4 peptide. Results are mean (*SEM*) of values obtained from 3 to 5 mice representative of 11–15 mice per group included in four separate experiments. One-way ANOVA followed by Tukey’s post hoc test was used for statistical analysis

Blood gas and electrolytes	Treatment Group						
	Untreated	Vehicle	0.625 μg/g bw^a^	1.25 μg/g bw	2.5 μg/g bw	5 μg/g bw	10 μg/g bw
pCO_2_ mmHg	54.00 (5.20)	64.00 (6.25)	58.50 (4.33)	42.67(11.39)	52.75 (6.51)	60.33 (5.24)	65.67 (4.70)
HCO_3_ mmol/L	22.88 (1.03)	22.47 (0.72)	23.18 (0.57)	23.30 (1.05)	22.98 (1.15)	21.73 (0.38)	23.57 (0.58)
tCO_2_ mmol/L	24.54 (1.18)	24.43 (0.90)	25.00 (0.50)	24.60 (0.70)	24.60 (1.28)	23.30 (0.21)	25.60 (0.69)
pO_2_ mmHg	29.20 (5.74)	34.00 (10.21)	27.50 (3.52)	35.67 (2.91)	37.50 (8.66)	38.67 (6.77)	24.67 (2.03)
tHB g/dL	15.20 (0.61)	14.03 (0.50)	15.00 (0.29)	12.03 (2.68)	14.18 (1.31)	11.43 (3.23)	15.27 (0.41)
Anion gap mmol/L	15.02 (0.38)	16.50 (0.85)	16.63 (1.15)	15.47 (2.49)	16.03 (1.09)	18.30 (0.76)	17.03 (0.42)
Base excess (mmol/L)	4.50 (0.21)	7.03 (0.35)	5.23 (1.42)	6.10^b^ (0.79)	4.13 (1.17)	7.27 (1.54)	6.10 (0.60)
Blood pH	7.27 (0.03)	7.19 (0.03)	7.24 (0.04)	7.42 (0.16)	7.29 (0.04)	7.20 (0.04)	7.20 (0.02)
Na^+^ mmol/L	145.4 (0.51)	147.3 (0.33)	148 (1.29)	147.7 (1.45)	146.3 (1.44)	148.0 (0.58)	148.0 (1.00)
K^+^ mmol/L	4.84 (0.21)	4.83 (0.69)	3.65 (0.17)	4.43 (0.59)	6.25 (1.33)	3.97 (0.18)	4.30 (0.12)
Cl^−^ mmol/L	112.0 (0.84)	113.3 (1.33)	111.8 (1.18)	113.3 (0.88)	112.8 (0.25)	111.7 (0.33)	111.7 (1.33)

^a^*bw* body weight, ^b^all values for base excess were negative except for one mouse

### Serum biochemistry profile of SPA4 peptide-treated mice

The levels of proteins (total protein, albumin, globulin), liver enzymes (ALP, ALT, GGT), bile acids and total bilirubin, analytes related to kidney function (BUN, creatinine), muscle enzymes (creatine kinase, AST, ALT), and glucose and cholesterol, associated with hormonal and metabolic imbalance, were determined in serum specimens collected at the time of necropsy. No measurable amounts of GGT and creatinine were detected in serum specimens of SPA4 peptide-treated mice. Total protein (range of means 4.5–5.2 g/dL), albumin (range of means 2.5–3.2 g/dL), and globulin (range of means 2.0–2.1 g/dL) levels did not significantly differ among study groups. Levels of liver enzymes (range of means 105.3–151.5 U/L ALP, 89–411 U/L AST, and 36.6–116.8 U/L ALT) remained statistically unaltered among different treatment groups. Levels of bile acids (range of means 3.7–18.9 μmol/L) were not different among treatment groups. Bilirubin levels varied (range of mean values 0.13–0.43 mg/dL) among different groups. One untreated mouse had an increased measured value of 1.9 mg/dL total bilirubin; the remaining untreated mice had 0.1–0.7 mg/dL total bilirubin. Thus, levels of total bilirubin in mice treated with 1.25 μg SPA4 peptide/g body weight (mean value 0.13 mg/dL) were noted statistically different from those in untreated mice (mean value 0.43 mg/dL). The levels of total bilirubin in other groups did not significantly differ from those in untreated mice.

**Fig. 5:**
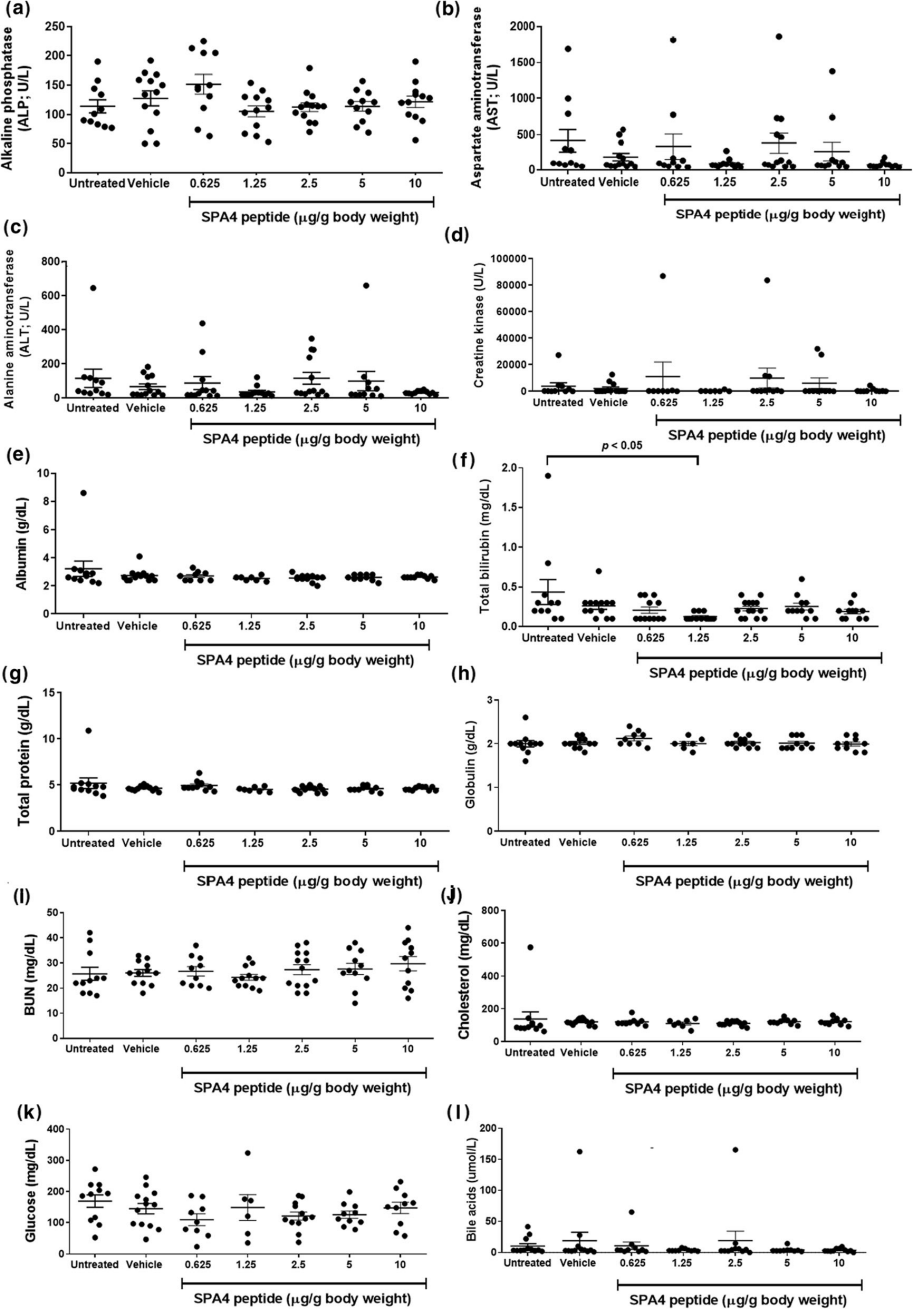
Serum biochemical profile of CD-1 mice treated with escalating doses of SPA4 peptide. Fresh blood harvested from mice in different groups were clotted at room temperature and subjected to separation of serum by centrifugation. Serum samples from 6 to 12 mice per group (seven groups total) were then analyzed for biochemical profile, specifically, ALP (**a**), AST (**b**), ALT (**c**), creatine kinase (**d**), albumin (**e**), total bilirubin (**f**), total protein (**g**), globulin (**h**), BUN (**i**), cholesterol (**j**), glucose (**k**), and bile acids (**l**). One-way ANOVA followed by Tukey’s post hoc analysis was used for statistical analysis of data from different study groups

Analytes related to kidney function (range of means 24.3–27.4 mg/dL BUN, no creatinine detected) and muscle enzymes [(range of means 330.1–11,084 U/L of creatine kinase), ALT, and AST] did not statistically differ among groups. Average measured levels of glucose (range of means 109.7–169.4 mg/dL) and cholesterol (range of means 110–137 mg/dL) were not significantly different among groups. No significant change in serum biochemical profile related to SPA4 peptide treatment indicated no liver, kidney, or cardiac injuries (Fig. 5).

### SPA4 peptide-specific IgG levels in mice

**Fig. 6:**
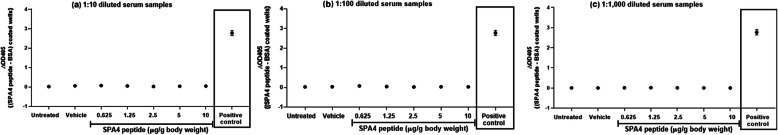
SPA4 peptide-specific IgG levels in serum samples harvested from SPA4 peptide-treated, vehicle-treated, and untreated CD-1 mice. Diluted mouse serum samples [1:10 (**a**), 1:100 (**b**), and 1:1000 (**c**)] were subjected to ELISA for detection of SPA4 peptide-specific IgG levels. The ∆OD405 readings for positive control wells are shown within the figure. The *SEM* bars smaller than the size of the symbol are not visible in the chart. There was no statistically significant difference in the ∆OD405 readings among different study groups (One-way ANOVA followed by Tukey’s post hoc analysis)

Results in Fig. 6 demonstrate that the ∆OD405 readings were not significantly different in 1:10, 1:100 and 1:1000 diluted serum samples from SPA4 peptide-treated, vehicle-treated and untreated mice (range of mean ∆OD405 readings < 0.0–0.075). Positive control well in each ELISA run included incubation with affinity-purified SPA4 peptide-specific antibody (range of mean ∆OD405 reading 2.774). Together, the results suggest that repeated treatment with SPA4 peptide did not induce significant IgG response.

### SPA4 peptide alleviates LPS-induced lung inflammation and injury

#### Levels of protein, albumin, lactate, lung edema, and symptoms of sickness

**Fig. 7:**
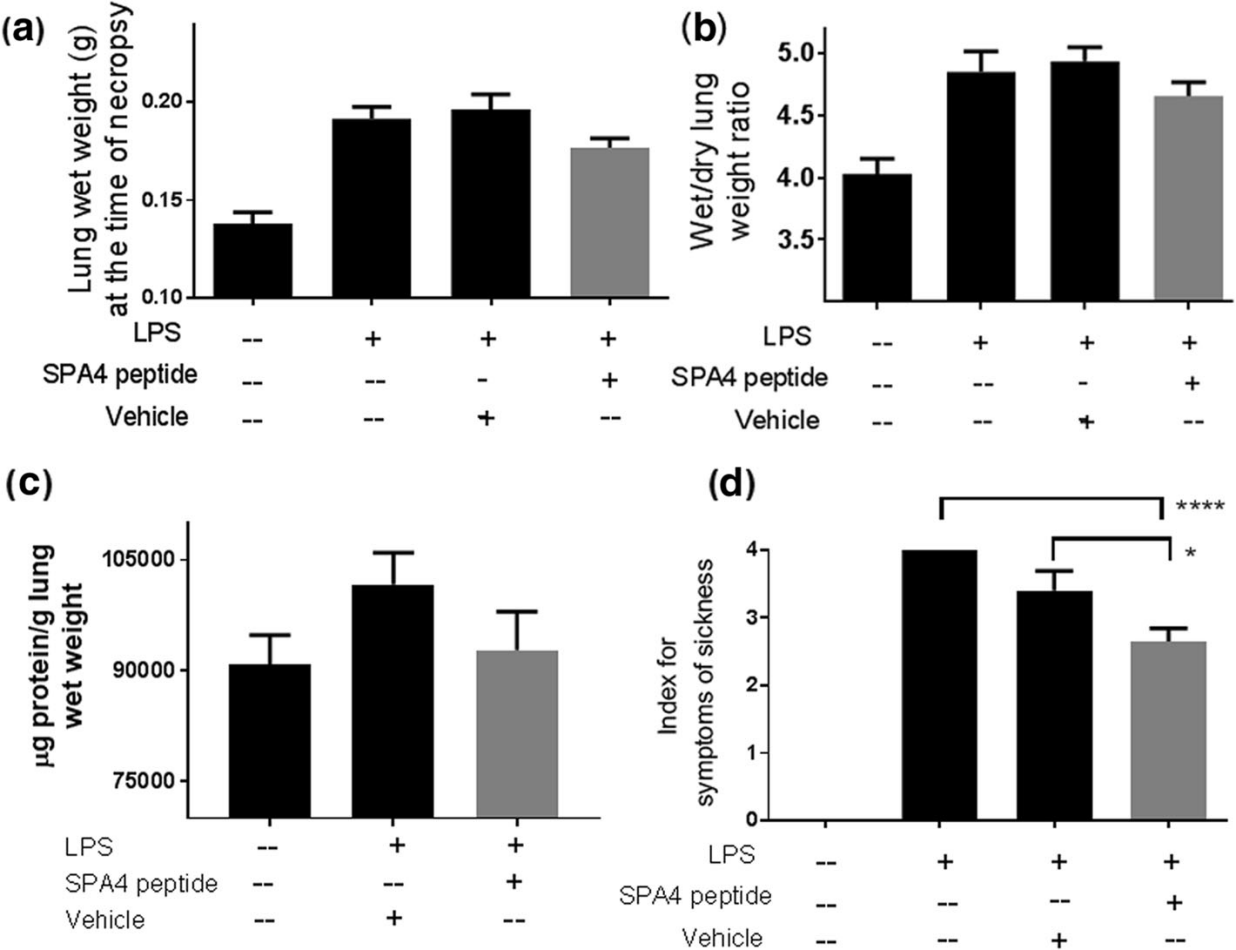
Effect of SPA4 peptide on LPS-induced lung edema and reactivity in an intratracheal LPS challenge model in C57BL6 mice. Wet weights of lungs were noted at the time of necropsy (**a**). Ratios of lung wet/dry weight (**b**), μg total protein/g lung wet weight (**c**), and index for the symptoms of sickness (**d**). Mean + *SEM* values of measurements are from 8 to 19 mice per group (lung wet weight), 3–15 mice per group (lung wet/dry weight ratio), 14–15 mice per group (μg protein/g lung wet weight), or 12–29 mice per group (symptoms of sickness) included in separate experiments. The *p* values (* *p* < 0.05, **** *p* < 0.0001) were determined by ANOVA with Tukey’s post-hoc analysis

Accumulation of fluid and inflammatory mediators resulted in an increase in the total lung protein, albumin, lactate, lung wet weight, and lung wet/dry weight ratio in LPS-challenged mice compared with unchallenged, untreated mice (Table 3 and Fig. 7). Evidence of edema was visually apparent, and an increase in lung wet weight was noted in LPS-challenged mice (Fig. 7). Correspondingly, in this acute and short-term model, we observed that the LPS-challenged mice were less reactive to tail-hold stimuli and showed mild signs of prostration. The SPA4 peptide treatment reduced the levels of lung protein, albumin, and lactate, lung wet weight, and lung wet/dry weight ratio, and alleviated the symptoms of sickness (Fig. 7).

**Table 3:** Blood gas and electrolyte parameters **(A),** and levels of lactate in serum and lung tissue homogenates **(B)**, and albumin in lung tissue homogenates **(C)** of LPS-challenged, vehicle-treated (LPS + vehicle); LPS-challenged, SPA4 peptide-treated (LPS + SPA4 peptide); and unchallenged and untreated C57BL6 mice. The numbers represent the mean (*SEM*) values derived from 6 to 16 mice per group**.** The *p* values (*p < 0.05, **p < 0.005, ***p < 0.0005, ****p < 0.0001 *versus* unchallenged, untreated; #*p* < 0.05 *versus* LPS + vehicle) were determined by ANOVA with Tukey’s post-hoc analysis

(A) **Blood gas and electrolytes**	**Unchallenged, untreated**	**LPS + vehicle**	**LPS + SPA4 peptide**
pCO_2_ mmHg	58.44 (3.28)	79.27 (3.77)**	74 (4.36)^*^
HCO_3_ mmol/L	24.52 (0.61)	28.25 (0.39)*	27.48 (1.49)
tCO_2_ mmol/L	26.3 (0.68)	30.68 (0.42)*	29.74 (1.60)
pO_2_ mmHg	37.67 (3.77)	49.2 (4.84)	43.25 (3.30)
tHB g/dL	15.12 (0.25)	15.02 (0.25)	14.35 (0.69)
AnGAP mmol/L	14.87 (0.26)	8.91 (0.53)*	11.76 (2.21)
Base Excess (mmol/L)	3.61 (0.63)	3.11 (0.61)	3.3 (1.20)^a^
Blood pH	7.267 (0.02)	7.199 (0.02)*	7.212 (0.01)
Na^+^ mmol/L	146.4 (0.53)	141.2 (0.52)****	142.6 (0.51)***
K^+^ mmol/L	4.84 (0.18)	4.10 (0.17)*	4.16 (0.17)^*^
Cl^−^ mmol/L	112.3 (0.49)	108.2 (0.66)^**^	108.2 (0.98)**
(B) **Lactate**	**Unchallenged, untreated**	**LPS + vehicle**	**LPS + SPA4 peptide**
In serum (pmol/μl)	3081 (190.3)	5647 (343.2)****	4684 (239.4)***, ^#^
In lung (nmol/g lung)	6931 (1646)	19,420 (7108)	10,439 (2248)
(C) **Albumin**	**Unchallenged, untreated**	**LPS + vehicle**	**LPS + SPA4 peptide**
In lung (μg/g lung)	10,525 (761.1)	15,882 (997.6)***	13,451 (708.8)

^a^all values for base excess were negative except for two mice

#### Lung inflammation indices

**Fig. 8:**
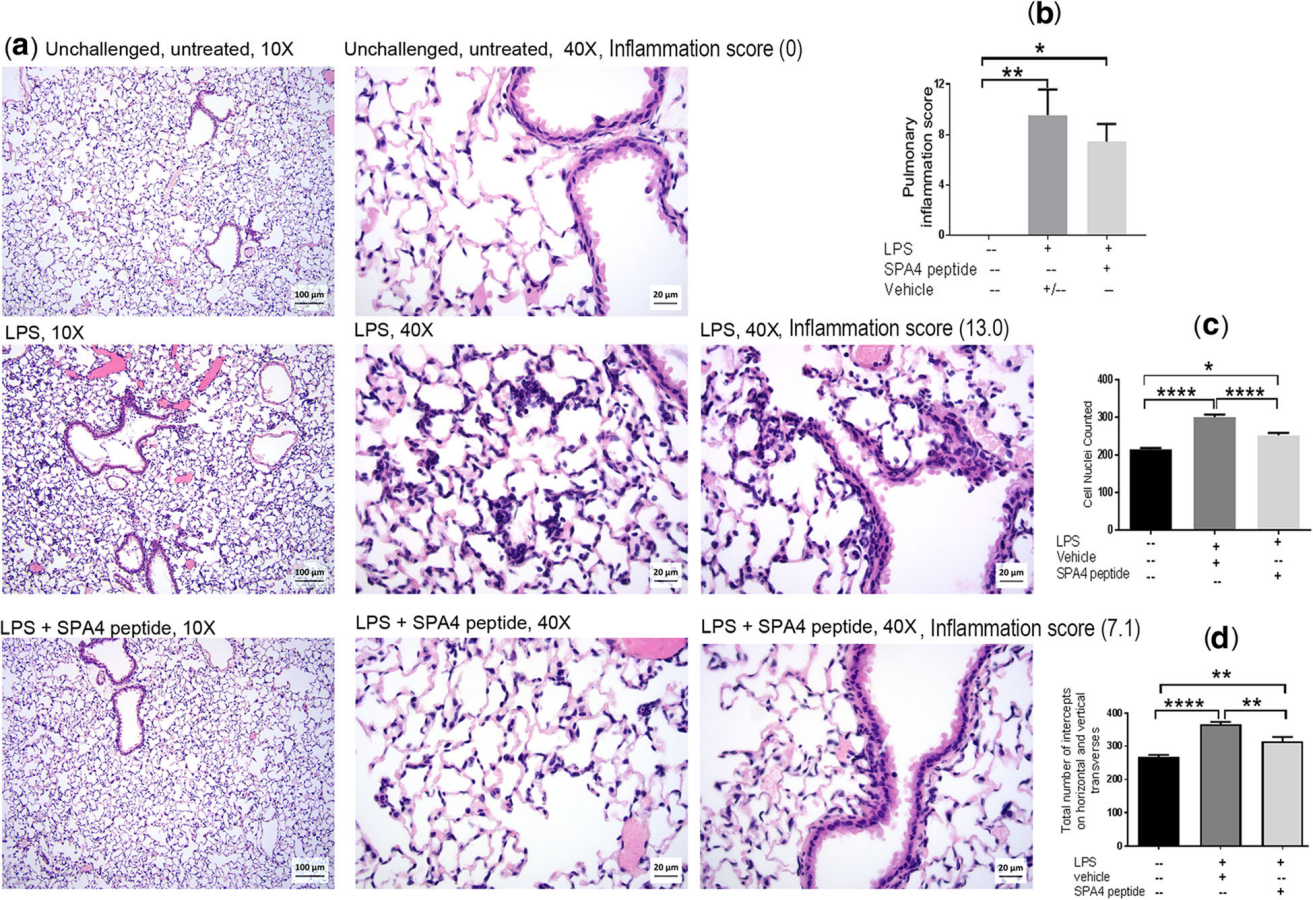
Assessment of inflammation in H&E-stained lung tissue sections of LPS-challenged and SPA4 peptide-treated C57BL6 mice. Representative photomicrographs of H&E-stained lung tissue sections of mice included in five separate experiments. The photomicrographs were taken using a 10X and 40X objective (**a**). Low and high magnification photomicrographs demonstrate differences in the semi-quantitative grading criteria, including the extent of peribronchial/bronchiolar infiltrates, severity of peribronchial/bronchiolar infiltrates, extent of alveolar infiltrates, and severity of alveolar septal and luminal neutrophilic infiltrates. The pulmonary inflammation scores (Mean + *SEM)* for mice in each group are shown as bar chart (**b**). Inflammation scores for individual representative images are indicated within (**a**). The number of cell nuclei counted in five randomly selected regions per lung tissue section (**c**). Total number of intercepts counted on horizontal and vertical transverses in five randomly selected regions per lung tissue section. The cell nuclei and number of intercepts were counted using the STEPanizer program (**d**). Mean + *SEM* values are shown within each figure for different types of measurements of lung inflammation. The *p* values (* *p* < 0.05, ** *p* < 0.005, **** *p* < 0.0001) were determined by ANOVA with Tukey’s post-hoc analysis

The intratracheal LPS challenge induced pulmonary inflammation, as determined by histopathological analysis of the extent and severity of inflammatory cell infiltrates, and microscopic analysis of cellularity and number of intercepts, in H&E-stained lung tissue sections. The SPA4 peptide treatment reduced the lung inflammation (average inflammation score 7.46 versus 9.58 in LPS-challenged mice; Fig. 8b).

As a measure of structural changes in the lung spaces, we counted the number of cells and linear intercepts in randomly selected regions of H&E-stained lung tissue sections. While selecting these regions, large airways and blood vessels were avoided. The SPA4 peptide treatment reduced the cellularity (Fig. 8c). A similar trend was obtained with the total number of intercepts counted on vertical and horizontal traverses in selected regions of photomicrographs (Fig. 8d). The cell nuclei and intercepts were counted by three independent blinded observers on different occasions.

#### Physiological parameters

Furthermore, we studied the levels of blood gas and electrolytes, and serum lactate levels as physiological measures. Our results showed that there were moderate to significant changes in the levels of these study parameters in LPS-challenged mice. SPA4 peptide significantly suppressed the LPS-induced serum lactate levels compared to those in vehicle-treated mice (Table 3). Similarly, the levels of other study parameters in SPA4 peptide-treated mice showed a trend towards the one noted in unchallenged, untreated mice.

## Discussion

The SPA4 peptide is derived from an endogenous protein, surfactant protein (SP)-A [2]. The SP-A is present in lung and in other mucosal organs, and is critical for maintenance of lung function and host defense. Decreased levels of SP-A, but increased expression levels of TLR4 are associated with lung diseases, including lung infection and inflammation [14, 15, 20]. Earlier, results have demonstrated that the SP-A exhibits pro-phagocytic and anti-inflammatory activity against bacterial stimuli through its interaction with TLR4 [21]. However, the pharmacological use of SP-A has not been possible. Although reasons are not completely known, large size and pro-inflammatory effects of SP-A related to its N-terminal region [22] could have contributed to its unsuitability for therapeutic application. Functionally relevant TLR4-interacting regions of SP-A, specifically SPA4 peptide, were delineated using advanced approaches.

The SPA4 peptide is a 20 mer hydrophilic peptide that interacts with TLR4 complex, and exhibits pro-phagocytic and anti-inflammatory activity against LPS and Gram-negative bacterial stimuli [3–6]. Initial results about the efficacy of SPA4 peptide are promising for its therapeutic effects in mouse models of lung inflammation and infection. The SPA4 peptide on its own does not induce an inflammatory response [2, 4, 6, 12]. Thus, the present work focused on assessing the health effects, toxicity profile, and biological activity of SPA4 peptide in mice.

The SPA4 peptide was evaluated for its toxic and health effects in CD-1 mice. An outbred CD-1 mouse strain was included because it exhibits genetic heterogeneity similar to humans, and is used widely for toxicity studies [9, 23]. An intratracheal instillation of SPA4 peptide was selected to determine the tissue-level toxicity in lung and systemic effects, and for its relevance in models of lung infection and inflammation [5]. Repeated intratracheal instillation was performed with SPA4 peptide to determine toxic effects over a total study period of 4 days. Both local lung and systemic toxicity parameters were included to obtain a comprehensive assessment of intratracheally administered SPA4 peptide. Mice were treated with the incremental doses of 0.625, 1.25, 2.5, 5, and 10 μg/g body weight of SPA4 peptide. A dose of 2.5 μg/g body weight of SPA4 peptide was comparable to those used in published reports on its biological activity in mouse models of lung infection and inflammation [4, 5], and in the intratracheal LPS challenge model described in this study. When calculated using an established formula [24, 25], human equivalent doses are 0.05, 0.1, 0.2, 0.4, and 0.8 mg/Kg for respective treatment doses in mice.

Biochemical parameters and edema were determined for an assessment of toxicity in lung. Specifically, the levels of total protein, albumin, and lactate, and activity of LDH were determined in the homogenates of harvested lung tissues from respective mice. Increased levels of total protein, albumin, and lactate indicate tissue damage and hypoxia. Total protein concentration also increases in injured lung [26]. It has been demonstrated that an increase in pulmonary release of lactate correlates with the severity of lung injury [27, 28]. Measurement of LDH activity in lung tissues has been used for an assessment of pulmonary toxicity [29]. An increase in the levels of albumin and LDH activity suggests potential membrane damage, leakage of cytoplasmic components, and cellular lysis, respectively [30]. There were no changes in the levels of total protein, albumin, and lactate, and LDH activity in lungs of SPA4 peptide-treated CD-1 mice (Fig. 2).

An accumulation of fluid leads to edema during lung injury; lung wet/dry weight ratio is used to assess the severity of pulmonary edema [31]. In the present study, there was no significant change in lung wet/dry weight ratios in different groups (Fig. 3). No adverse effects were observed on lung toxicity parameters (levels of total protein, albumin, and lactate, activity of LDH, and lung wet/dry weight ratio), even at the maximum dose of 10 μg SPA4 peptide/g body weight included in this study.

Histologically, tissue specimens harvested from most mice revealed normal anatomical and structural features (Fig. 4, Table 1). Signs of mild damage were observed in kidney, lung, and liver tissue specimens from one mouse out of a total of 11–15 mice per group in selected study groups (Table 1), which were not related to the intratracheal route of treatment, dose of administered SPA4 peptide, or study design. These changes are most likely due to genetic and phenotypic variations reported in CD-1 outbred mice [7].

Altered vasculature during acute lung injury leads to leakage of metabolites and chemical mediators into circulation, which causes systemic effects, hypoxia in distal organs, and organ failure. Thus, systemic effects of SPA4 peptide were studied by evaluating the serum biochemical profile and levels of blood gas and electrolytes. Serum biochemistry profiles of SPA4 peptide-treated mice demonstrated no change in biochemical markers of liver (serum bilirubin, ALT, AST, ALP, GGT), kidney (serum creatinine and BUN), or cardiac (creatine kinase, blood gas, and electrolytes) injuries, compared with those in untreated or vehicle-treated mice (Fig. 5, Table 2). Most of the read-outs for different analytes were consistent, except for total bilirubin level in one untreated mouse, which was not related to SPA4 peptide treatment.

The read-outs of blood gas and electrolyte levels were compared for an analysis of overall physiology and metabolism in SPA4 peptide-treated mice (Table 2). Independently, when pH values were calculated by Henderson-Hasselbach equation using measured values of blood pCO_2_ and HCO_3_^−^ [32], the calculated values were similar to the measured values of blood pH in mice among different study groups. We identify that the isoflurane anesthesia and collection of venous or mixture of venous and arterial blood by cardiac puncture may have affected the basal values of pO_2_ and pCO_2_, as reported by other researchers [33–35]. However, there was no statistically significant change in the measured levels of blood gases (pCO_2_ and pO_2_) and electrolytes in mice among different study groups (Table 2). These results in conjunction with other study parameters, suggest no major sign of respiratory, metabolic, or kidney disorders in SPA4 peptide-treated mice. Other published research studies have also investigated parameters of toxicity in other non-pulmonary organs of animals intratracheally treated with different agents, such as statin [36], vascular endothelial growth factor-small-interfering RNA [37], iron oxide and bismuth selenide nanoparticles [38, 39], ,and amphotericin B formulation [40].

In order to assess the immune response that can arise from SPA4 peptide treatment, the SPA4 peptide-specific total IgG antibody levels were measured in serum samples (Fig. 6). In this short-term repeated treatment model, SPA4 peptide-specific IgG antibody levels were not detectable in serum samples from any of the study groups, including the mice treated with the maximum dose of SPA4 peptide. Several factors, including mouse strain, can influence the immunogenicity, the results presented here provide an initial assessment.

Simultaneously, we determined the biological activity of intratracheally administered SPA4 peptide in an intratracheal LPS challenge model of lung inflammation in C57BL6 mice (Figs. 7, 8, and Table 3). In our previously published report, we determined the extent of lung inflammation in a mouse model of endotoxic shock induced by intraperitoneal injection of 15 μg LPS/g body weight [4]. While the intraperitoneal LPS challenge model represents systemic injury, the intratracheal LPS challenge mimics local inflammation in the lung. As such, lungs could be constantly exposed to some amounts of LPS present as a contaminant on airborne particles, organic dusts, and cigarette smoke [41]. Studies in intratracheal LPS challenge model could potentially be more relevant to lung conditions. Moreover, the intratracheal LPS challenge model also represents the disease condition of Gram-negative bacterial lung infection in which the bacteria are already cleared, but significant amounts of LPS are present and cause inflammation. The challenge dose of LPS (5 μg/g body weight, one mouse weighing about 20 g; 100 μg LPS per mouse) induces an inflammatory response in the lungs within 4 h, and is relevant to the biological scenarios.

Our results demonstrate that the SPA4 peptide reduces the LPS-induced lung edema, as evidenced by reduction in lung wet weight, total protein, and lung wet/dry weight ratio (Fig. 7). Reduced lung edema corresponds with improvement in reactivity of mice to tail-hold stimuli and prostration (Fig. 7). Correspondingly, the cellularity in lung tissue sections was affected. These results were further supported by reduced lung inflammation scores in SPA4 peptide-treated mice (Fig. 8). Reduced inflammation in the lungs corroborated with changes in blood gas and electrolytes levels; the trend in SPA4 peptide-treated mice is close to the one noted for unchallenged, untreated healthy mice (Table 3). The levels of lactate, albumin, and total protein were reduced in lung tissue homogenates of SPA4 peptide-treated versus vehicle-treated mice against LPS stimuli (Table 3 and Fig. 7). Serum lactate levels, indicator of severe tissue injury, were significantly reduced in SPA4 peptide-treated mice (Table 3). Together, the work presented here provides further evidence of the anti-inflammatory effects of SPA4 peptide in a mouse model of inflammation induced by LPS challenge. These results are consistent with the reported effectiveness of intraperitoneally administered SPA4 peptide in reducing inflammation in a mouse model of intraperitoneal LPS-challenge [4]. The maximum dose of 10 μg SPA4 peptide/g body weight, included in the toxicity study in CD-1 mice, is about four times higher than what was used in published efficacy studies in mouse models of lung infection and inflammation [4, 5], and in mouse model of lung inflammation described here (Figs. 7, 8, and Table 3). The results presented here suggest that the intratracheal administration of SPA4 peptide does not induce any toxic effect at cellular, tissue, and organ levels, but exerts its anti-inflammatory activity through its binding to TLR4 activated by LPS stimuli and immunomodulatory mechanism(s). Although the mechanism(s) of action of SPA4 peptide is not completely known at this time, the results presented here provide an initial assessment of a toxicity profile, substantiate the anti-inflammatory activity, and contribute to understanding of its basic immunobiology.

## Conclusions

The prevalence of pulmonary toxicity is high with many drugs which can be progressive and fatal. Early recognition of any toxicity of any new therapeutic candidate is important. This is the first evidence of the nontoxic effect of SPA4 peptide at local and systemic levels. Since clinical surfactant does not contain SP-A or its components, it is expected that the inclusion of SPA4 peptide could enhance the efficacy of these formulations used for the lung conditions of prematurely delivered infants or adults. Intratracheal administration is used to deliver drugs into the lungs of patients suffering from debilitating pulmonary disease conditions. The lack of observed toxic effect by SPA4 peptide instillation and its efficacy in lung infection and inflammation point towards its potential application in critically ill patients, who are most commonly intubated for treatment in the intensive care units. In conclusion, the study findings provide a solid rationale for conducting comprehensive pre-clinical safety and toxicity assessment of SPA4 peptide in future. Furthermore, an assessment of biological activity of intratracheally administered SPA4] peptide in intratracheal LPS-challenge model substantiates its anti-inflammatory activity. It is expected that the SPA4 peptide treatment will exert anti-inflammatory activity without causing any significant toxic effects in lung or other non-pulmonary organs in the body of the host.

## Data Availability

All the data are included in this article.

## Abbreviations

AG: Anion gap
ALP: Alkaline phosphatase
ALT: Alanine aminotransferase
ANOVA: Analysis of variance
AST: Aspartate aminotransferase
BCA: Bicinchoninic acid
BE: Base excess
BSA: Bovine serum albumin
BUN: Blood urea nitrogen
Cl^−^: Chloride
EDTA: Ethylenediamine tetraacetic acid
ELISA: Enzyme-linked immunosorbent assay
GGT: Gamma-glutamyl transferase
HCO_3_^−^: Bicarbonate
H&E: Hematoxylin and eosin
HPF: High-power field
HPLC: High-performance liquid chromatography
IgG: Immunoglobulin G
K^+^: Potassium
LDH: Lactate dehydrogenase
LPS: Lipopolysaccharide
Na^+^: Sodium
NAD: Nicotinamide adenine dinucleotide
OD: Optical density
OUHSC: University of Oklahoma Health Sciences Center
pCO_2_: Partial pressure carbon dioxide
pH: Hydrogen ion concentration
pO_2_: Partial pressure oxygen
RNA: Ribonucleic acid
SP-A: Surfactant protein-A
TBST: Tris buffered saline containing 0.05% Tween 20
tCO_2_: Total carbon dioxide
tHb: Total hemoglobin concentration
TLR4: Toll-like receptor 4
